# Ki-67 Labelling Index as a Predictor of Invasive Features in Thyroid Cancer: Retrospective Analysis and Implications

**DOI:** 10.3390/curroncol31070300

**Published:** 2024-07-17

**Authors:** Raisa Chowdhury, Raihanah Alsayegh, Véronique-Isabelle Forest, Marc Philippe Pusztaszeri, Sabrina Daniela da Silva, Livia Florianova, Richard J. Payne

**Affiliations:** 1 Faculty of Medicine, McGill University, Montreal, QC H3G 2M1, Canada; 2 Department of Otolaryngology-Head and Neck Surgery, McGill University, Royal Victoria Hospital, Montreal, QC H4A 3J1, Canada; 3 Department of Otolaryngology-Head and Neck Surgery, McGill University, Jewish General Hospital, Montreal, QC H3T 1E2, Canada; 4 Department of Pathology, McGill University, Jewish General Hospital, Montreal, QC H3T 1E2, Canada

**Keywords:** thyroid cancer, Ki-67 labelling index, aggressiveness, prognostic marker, histological subtypes

## Abstract

Background: Ki-67 immunostaining is commonly used in neuroendocrine tumors to estimate the proliferative index and for grading. This study investigates its association with the invasiveness of follicular-derived thyroid carcinomas (TCs). Methods: A retrospective analysis of patients with TC at three McGill University teaching hospitals between January 2018 and November 2023 was conducted. The inclusion criteria included patients with malignant thyroid tumors and accessible Ki-67 LI data from final pathology specimens. The data collected included patient demographics, Ki-67 LI values, and different invasiveness attributes, such as molecular mutations, the histological subtype, lymphovascular invasion (LVI), extrathyroidal extension (ETE), and positive lymph nodes (LNs). Results: In total, 212 patients met the inclusion criteria, of which 80.7% were females and 19.3% were males. The Ki-67 LI ranged from 1% to 30%, with the majority of the cases within the range of 1–15%. A significant association was observed between higher Ki-67 LI and high-risk histological subtypes of thyroid carcinoma (*p* < 0.001). Similarly, Ki-67 LI was significantly associated with LVI and positive LN metastasis (*p* < 0.001 and *p* = 0.036, respectively). However, no significant association was found between the Ki-67 LI and gene mutations or ETE (*p* = 0.133 and *p* = 0.190, respectively). Using percentiles to establish a cutoff, patients with a Ki-67 LI higher than 6.7 showed a higher likelihood of being associated with invasive features. Conclusion: Elevated Ki-67 LI can serve as an indicator of aggressiveness in follicular-derived TC, especially when associated with distinct histological subtypes, LVI and positive LNs.

## 1. Introduction

Thyroid cancer (TC), recognized as the predominant malignancy within the endocrine system, has shown a consistent increase in incidence over recent decades. Papillary thyroid carcinoma (PTC) and follicular thyroid carcinoma (FTC), also called differentiated thyroid carcinomas (DTCs), account for nearly 95% of diagnosed malignancies [1,2]. Prognostic indicators in TC such as clinicopathological factors, molecular biomarkers and cellular markers play a critical role in customizing treatment and monitoring postoperatively for recurrent disease [3,4,5].

Utilizing immunohistochemistry methodologies, Ki-67, a protein indicative of cellular proliferation, staining facilitates the determination of the proportion of Ki-67-positive cells, providing insights into the proliferative vigor of the tissues under study [6]. The Ki-67 labelling index (LI) is defined as the percentage of Ki-67-antigen-positive cells. Studies on Ki-67 LI in DTC present conflicting evidence regarding its correlation with invasive traits such as the tumor size, lymph node involvement, and extrathyroidal extension, resulting in varied interpretations and inconclusive findings [7]. Discrepancies in these findings arise from differences in the study criteria, methodologies, and the use of diverse markers and definitions for invasiveness. This conflicting landscape underscores the imperative need for further investigations aimed at establishing a definitive relationship between Ki-67 expression and the manifestation of invasive characteristics in DTC [8]. A high Ki-67 LI (>10%) has been associated with tumor progression, poor disease-free survival and increased mortality in TC patients [9].

The primary objective of this study was to assess the potential correlation between elevated Ki-67 LI and invasiveness in follicular-cell-derived TC. The secondary objectives include exploring specific TC subtypes where this association may be more pronounced.

## 2. Materials and Methods

This retrospective analysis involves a cohort of 212 patients at three McGill University teaching hospitals undergoing thyroid surgery spanning over a five-year period from January 2018 to November 2023. The inclusion criteria include patients diagnosed with malignant DTC and having Ki-67 LI in their final pathology specimens. The exclusion criteria included all benign nodules and TCs that fall outside of DTC or PDTC, as well as any DTC that did not have information on Ki-67 LI in the pathology report. Data acquisition involved a comprehensive compilation of patient demographics alongside quantitative Ki-67 values. Additionally, detailed information regarding the histopathological attributes indicative of invasiveness was systematically extracted from the pathology reports. Invasive features were defined by the presence of one or more of the following: high-risk molecular mutations and fusions (e.g., *BRAF V600E*, *TERT* promoter, *TP53*, *NTRK* fusion), high-risk histological subtypes (tall cell, hobnail, diffuse sclerosing, and columnar cell PTCs, PDTC), lymph-vascular invasion (LVI), extrathyroidal extension (ETE), and lymph node (LN) involvement. These criteria formed the basis for exploring the associations between Ki-67 LI and the defining characteristics of aggressive behavior in TC cases. 

**Figure 1:**
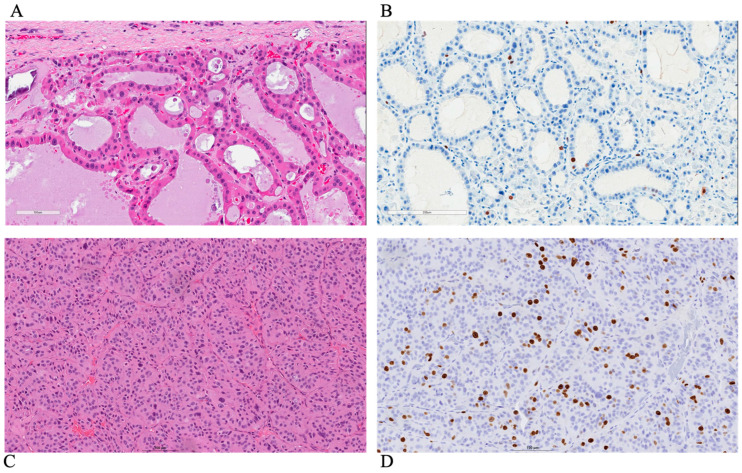
Representative pathology and Ki-67 immunostaining images of thyroid carcinomas: (**A**,**B**) Papillary thyroid carcinoma of the oncocytic subtype with a follicular architecture, showing a low Ki-67 proliferative index (1–2%). (**C**,**D**) Poorly differentiated thyroid carcinoma of a oncocytic subtype with a solid/trabecular architecture, showing an elevated Ki-67 proliferative index (around 15%).

The primary antibody used for this evaluation was Ki-67 [10]. All Ki-67 staining was performed on the resection specimens, and no cell blocks or fine needle aspiration (FNA) materials were used. The Ki-67 rate was determined quantitatively by manual counting. Representative pictures of the pathology and Ki-67 immunostains for high-grade differentiated and dedifferentiated thyroid carcinoma are shown in Figure 1. In particular, Ki-67 LI was defined as the percentage of immunopositive tumor cells (nuclear staining only) in areas showing the highest proliferative activity (so-called hotspots) in 500–2000 tumor cells, as described in other studies [11,12].

Descriptive analysis was performed to summarize the distribution of Ki-67 LI among different subgroups based on invasiveness markers such as mutations, histologic types, LVI, ETE and LN involvement. The measure data were described as mean ± standard deviation (SD). Data analysis by ANOVA was required to prove the homogeneity of variance from normally distributed data. If one of the above conditions was not met, a non-parametric test could be used instead. The significance level was set as *p* < 0.05. Statistical analyses were performed using STATA^®^ (STATA Corp., College Station, TX, USA). The study adhered strictly to ethical guidelines and was conducted following approval from the Research Ethics Board (REB) (2024-3826) of the respective McGill University teaching hospitals. All patient information was anonymized, ensuring strict adherence to patient privacy regulations such as the Health Insurance Portability and Accountability Act (HIPAA) guidelines.

## 3. Results

### 3.1. Demographic Characteristics

Among the 212 patients included in this study, 80.7% were female, while 19.3% were males. The age distribution was broad, ranging from 17 to 86 years, with an average age of 51.1 years and a median of 50 years. The SD was around 14.1 years, reflecting a wide age variation.

### 3.2. Ki-67 LI

The Ki-67 LI ranged from 1 to 30%, with the majority of the cases showing an LI within the range of 1–15% (Table 1).

### 3.3. Mutational Profile and High Risk

**Figure 2:**
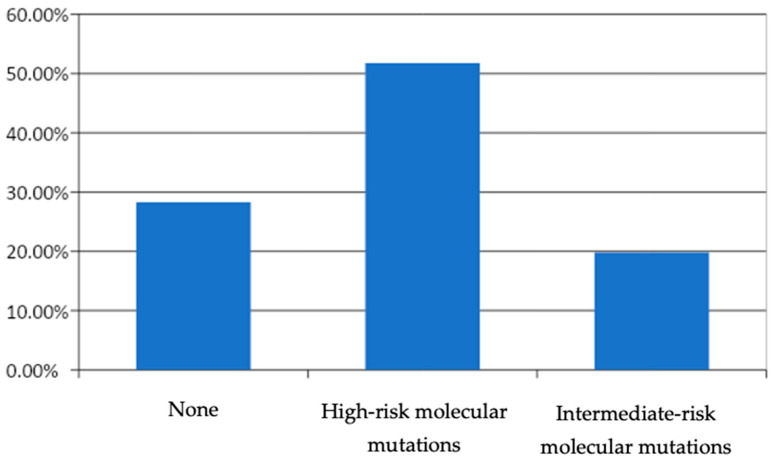
Mutation distribution. This figure shows the distribution of mutations across the study cohort, including high-risk and intermediate-risk mutations. High-risk mutations include *BRAF V600E* and *TERT*, while intermediate-risk mutations include *NRAS*, *HRAS*, *TSHR*, and *PTEN*.

The mutational profile revealed that 48.1% of cases were positive for mutations associated with a high risk (*BRAF*-like), among which the *BRAF V600E* variant showed the highest prevalence (44.8%). Twenty percent showed mutations of intermediate risk (RAS-like), including *NRAS* (5%), *EIF1AX* (4%), *HRAS* (3%), *TSHR* (1.5%), and *PTEN* (1%); meanwhile, 28.3% showed no mutations (Figure 2).

### 3.4. Association between Ki-67 LI and Mutational Landscape

High-risk (*BRAF*-like) mutations showed a slightly but not significatively elevated Ki-67 LI (6.23%, SD = 5.034) compared to those with no mutations or intermediate-risk mutations (5.27%, SD = 4.124), (*p* = 0.133) (Table 2).

### 3.5. Histological Classifications

The most prevalent subtype was the papillary carcinoma follicular variant (29.7%), followed by the papillary carcinoma classical (25.45%) and papillary carcinoma tall-cell variant (17.45%).

### 3.6. Correlation with Histological Subtypes

The analysis examined the relationship between different histological subtypes and the levels of Ki-67 LI. A higher Ki-67 LI was observed in the high-risk subtypes (8.39%, SD = 5.502) than in the non-invasive subtypes (4.55%, SD = 3.582); the association was significant (*p* < 0.001) (Table 2).

### 3.7. Ki-67 Labeling Index and LVI

The analysis revealed that the mean Ki-67 LI was higher in cases with LVI (10.11%, SD = 6.698) compared to those without LVI (4.90%, SD = 3.537); the association was significant (*p* < 0.001). The maximum Ki-67 LI in the LVI-positive group was also high (30%) compared to the non-LVI group (20%) (Table 2).

### 3.8. Ki-67 LI and LN

The results showed that cases with LN involvement have a higher mean Ki-67 LI (6.86%, SD = 4.406) than those without LN involvement (5.36%, SD = 4.657); the association was significant (*p* = 0.036) (Table 2).

### 3.9. Ki-67 LI and ETE

The results showed no significant association between the two parameters (*p* = 0.334) (Table 2).

### 3.10. Ki-67 LI Cutoff

**Figure 3:**
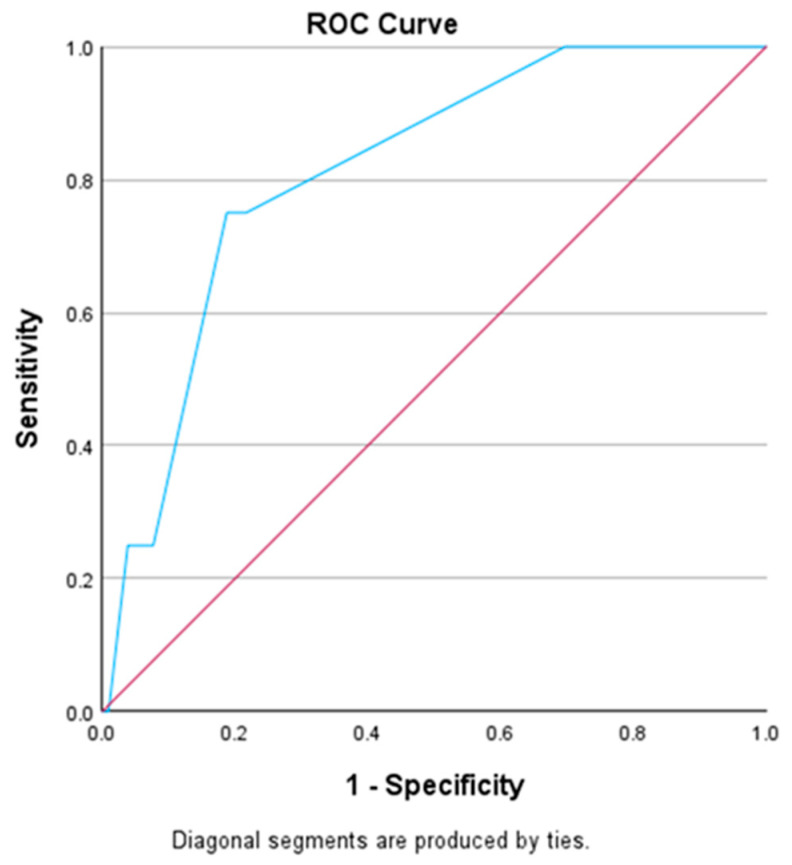
ROC curve for Ki-67.

Using percentiles to establish a cutoff in our study provided a clinically relevant way of determining the effectiveness of the markers that are being testing related to invasiveness. Patients with a Ki-67 LI higher than 6.7 showed a higher likelihood of being associated with invasive findings. (Table 1). The ROC curve in Figure 3 shows the best balance between the sensitivity and specificity of our test marker/classifier, in our case considering the invasion status.

## 4. Discussion

The study revealed a strong association between elevated Ki-67 LI and some invasive features in TC, including histological subtypes, LVI, and LN. These findings suggest Ki-67’s potential as a prognostic marker for TC. It was previously demonstrated in a study of 371 papillary carcinoma patients that the Ki-67 LI is an independent predictive factor for disease-free survival and that patients with high Ki-67 LI values have a noticeably lower disease-specific survival than patients with low Ki-67 LI values [13]. In a separate study conducted by Ziad et al. (2008), a notable increase in the Ki-67 LI within anaplastic areas compared to their follicular counterparts was observed, highlighting distinct proliferation rates in different segments of these complex thyroid tumors [14]. Another study by Harahap et al. (2022) showed that the Ki-67 LI is associated with an elevated likelihood of recurrence [15]. Moreover, Matsuse et al. (2017) demonstrated that a high Ki-67 LI is associated with higher recurrence rates and worse recurrence-free survival in PTC patients [16]. Tang et al.‘s study (2018) revealed a substantial association between a high Ki-67 LI and various factors in PTC, such as a larger tumor size, coexisting thyroiditis, and poorer disease-free survival [7]. Additionally, Moreas et al. (2008) observed that a heightened Ki-67 LI correlated with an invasive phenotype in PTC cases [17]. In contrast, Müller-Höcker’s (1999) research, which focused on oncocytic neoplasms, highlighted a significantly elevated Ki-67 LI in oncocytic carcinomas compared to oncocytic adenomas [18]. Furthermore, Hellgren et al. (2022) demonstrated that the Ki-67 labeling index was significantly higher in FTCs compared to FTAs, with mean values of 5.8% and 2.6%, respectively [19]. They established a cut-off value of 4% for the Ki-67 labeling index to differentiate FTCs from FTAs, with a sensitivity of 65% and specificity of 83% [19]. Their study also identified that the Ki-67 labeling index is an independent predictor of metastasis/recurrence and death in FTCs, with a higher risk of poor outcomes associated with Ki-67 indices greater than 4% [19]. These collective findings suggest that Ki-67 may serve as an indicator of invasiveness, particularly in PTC and FTC. However, a comprehensive understanding of the relationship between Ki-67 and adverse factors across different types of TC requires further exploration. 

Moreover, the integration of Ki-67 and molecular markers such as *BRAF V600E* could potentially enhance prognostic precision. According to Tufano et al. (2012), papillary thyroid cancer with lymphocytic infiltrates is more likely to have the *BRAF V600E* mutation [20]. In a correlation analysis between the *BRAF* mutation, Ki-67 expression, their combination and various clinical and pathological parameters in PTC patients, older age was positively correlated with a higher frequency of the *BRAF V600E* mutation (r = 0.284; *p* < 0.001), while a more advanced tumor stage was positively correlated with a higher Ki-67 LI (r = 0.307; *p* < 0.001) [20]. Furthermore, the tumor size, bilateral tumor site, multifocality, extracapsular invasion, and lateral LNM were strongly correlated with the *BRAFV600E* mutation and the *BANCR* (*BRAF*-activated non-coding RNA) and *miR-9* expression. To lower the risk of recurrence in PTC, patients with the *BRAFV600E* mutation, high *BANCR* expression, and low miR-9 expression, had earlier surgical therapy required and it is advised that a total thyroidectomy is undertaken during the first surgery [21]. Another systematic review identified the following significant risk factors for the *BRAFV600E* mutation in PTC patients: age (≥45 years), gender (male), multifocality, LNM, vascular invasion, extrathyroidal extension, and the advanced tumor node metastasis stage (stages III and IV). Tumor size (>1 cm) and distant metastasis do not appear to be correlated with the *BRAFV600E* mutation in PTC patients [9,13,15,22,23]. 

In thyroid cancer, determining a cutoff point for the Ki-67 LI is important for prognosis and treatment decisions. Analyzing data from 212 patients, we found that the Ki-67 LI varied from 0 to 30%. A clinically meaningful cutoff value of 6.7% was established through statistical analysis to support our study’s conclusions regarding the relationship between invasive thyroid tumors and the Ki-67 LI. Patients with Ki-67 levels over this cutoff showed a greater likelihood of invasive disease, which is in line with the ROC curve analysis evaluating sensitivity and specificity.

Previous studies have established the role of Ki-67 in predicting disease-free survival and the recurrence rates in various thyroid carcinoma subtypes. Our study focuses on the correlation of Ki-67 LI with invasiveness in follicular-derived thyroid carcinoma (FTC), an area less explored compared to papillary thyroid carcinoma (PTC). This work contributes to the existing literature by offering a detailed statistical analysis, enhancing the reliability and generalizability of our findings. 

The main limitation of this study is its retrospective nature. Additionally, the Ki-67 evaluation was not automated, which may have affected the results due to various preanalytical and analytical factors. Future studies should focus on larger and more diverse groups to confirm the results and determine the exact Ki-67 cut-off value for prognostic prediction, ideally using digital pathology and automated methods. Extending the follow-up periods, exploring combined markers such as *BRAF V600E*, and investigating the mechanisms of Ki-67 in various cancer subtypes can offer greater insights and improve prognostic accuracy. Exploring Ki-67’s ability to predict responses to targeted therapies could impact personalized treatments for thyroid cancer.

## 5. Conclusions

In conclusion, this comprehensive analysis of various factors of invasiveness in relation to the Ki-67 LI in TC has yielded valuable insights. Tumors with a higher Ki-67 LI were found to be associated with invasive histological subtypes of TC, LVI, and positive LNs, underscoring its role as a potential prognostic marker. 

## Figures and Tables

**Table 1:** Summary of key characteristics in the study cohort of thyroid cancer patients.

Variable	Frequency (N)	Percent (%)
**Ki-67 LI**		
- 1%	28	13.2
- 2%	21	9.9
- 3%	12	5.7
- 4%	2	0.9
- 5%	101	47.6
- 7%	4	1.9
- 8%	2	0.9
- 10%	25	11.8
- 12%	2	0.9
- 15%	6	2.8
- 20%	7	3.3
- 25%	1	0.5
- 30%	1	0.5
**Percentiles**	25	2.88
	50	4.85
	75	6.73

**Table 2:** Association between Ki-67 expression and relevant variables in thyroid cancer patients.

Variable	Group	Mean Ki-67 (%)	*p*-Value
High-Risk Mutations	No	5.27	0.133
	Yes	6.23	
Aggressive Subtypes	No	4.55	<0.001
	Yes	8.39	
Positive for LVI	No	4.90	<0.001
	Yes	10.11	
Positive for LN Involvement	No	5.36	0.036
	Yes	6.86	
Positive for ETE	No	5.73	0.334
	Yes	8.33	

Association table presenting the mean Ki-67 expression (%) for different categories of variables. The invasiveness of the mutations and histological, LVI, LN, and ETE are categorized as 0 (No) and 1 (Yes). *p*-values indicate the significance levels of the associations.

## Data Availability

The data presented in this study are available upon request from the corresponding author. The data are not publicly available due to the ethics approval agreement.
